# Supplementary material for: PIGMENT: A deep learning framework for Porcine Immunohistochemistry seGMENTation
Source: bioRxiv. 2026 Jun 29:2026.06.18.733245. Preprint. [Version 2] doi: 10.64898/2026.06.18.733245 (PMC13320970; doi:10.64898/2026.06.18.733245)
Supplement: Supplement 1 [file NIHPP2026.06.18.733245v2-supplement-1.pdf]

bioRxiv preprint doi: https://doi.org/10.64898/2026.06.18.733245; this version posted June 29, 2026. The copyright holder for this preprint (which was not certified by peer review) is the author/funder, who has granted bioRxiv a license to display the preprint in perpetuity. It is made available under a CC-BY 4.0 International license.

Supplementary Information

Fig. S1.

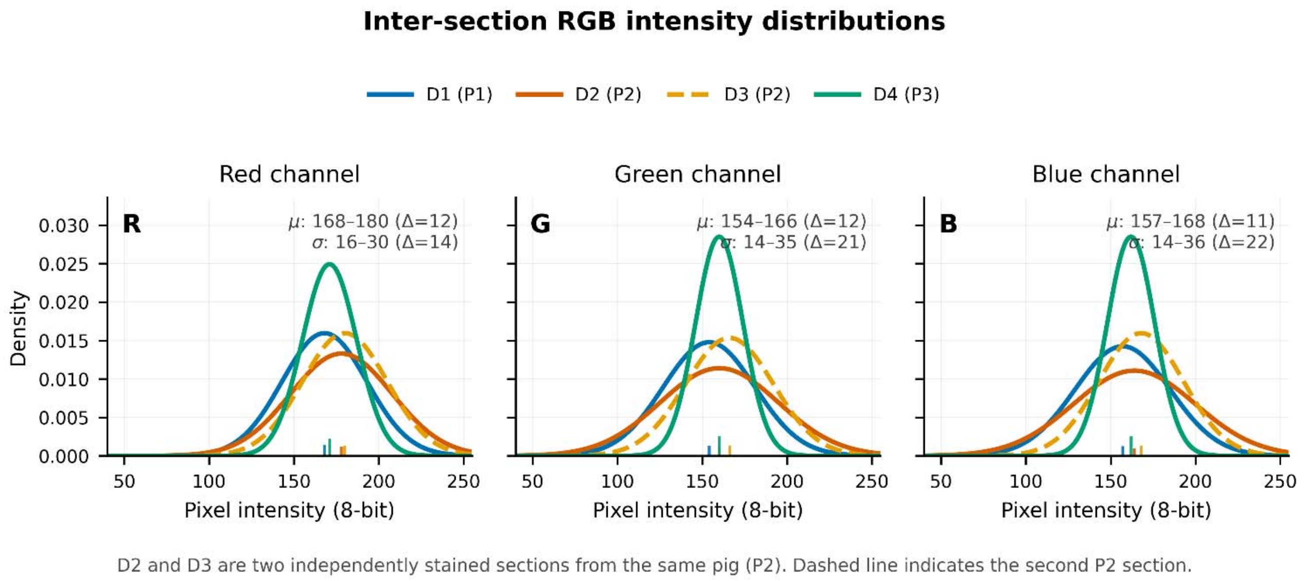

Figure S 1 Inter-section RGB intensity distributions across APP-stained porcine white-matter sections. Gaussian approximations of 8-bit red, green, and blue channel intensity distributions are shown for the four sections used in the PIGMENT dataset. Each panel overlays the same four sections for one color channel, allowing direct comparison of staining and acquisition variability across sections. D2 and D3 are independently stained sections from the same pig, with D3 shown as a dashed line. Short vertical ticks indicate channel-specific mean intensities. Differences in curve position reflect inter-section shifts in color intensity, whereas differences in curve width reflect variation in within-section intensity dispersion. These batch-level differences motivate the photometric augmentation strategy used during PIGMENT training.

Fig. S2.

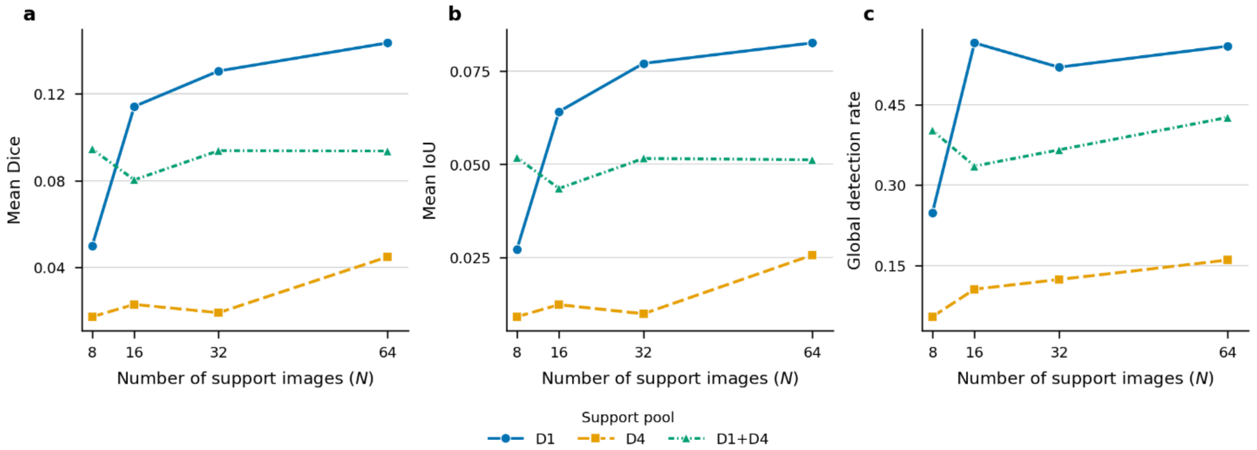

Figure S 2 Dice, IoU, and DR for the PIGMENT model with geometric augmentations across the four training configurations. The pattern matches Fig. 4 (full pipeline): DR is consistently higher than pixel-overlap metrics, and the D1+D4 configuration is strongest overall.

bioRxiv preprint doi: https://doi.org/10.64898/2026.06.18.733245; this version posted June 29, 2026. The copyright holder for this preprint (which was not certified by peer review) is the author/funder, who has granted bioRxiv a license to display the preprint in perpetuity. It is made available under a CC-BY 4.0 International license.

Fig. S3.

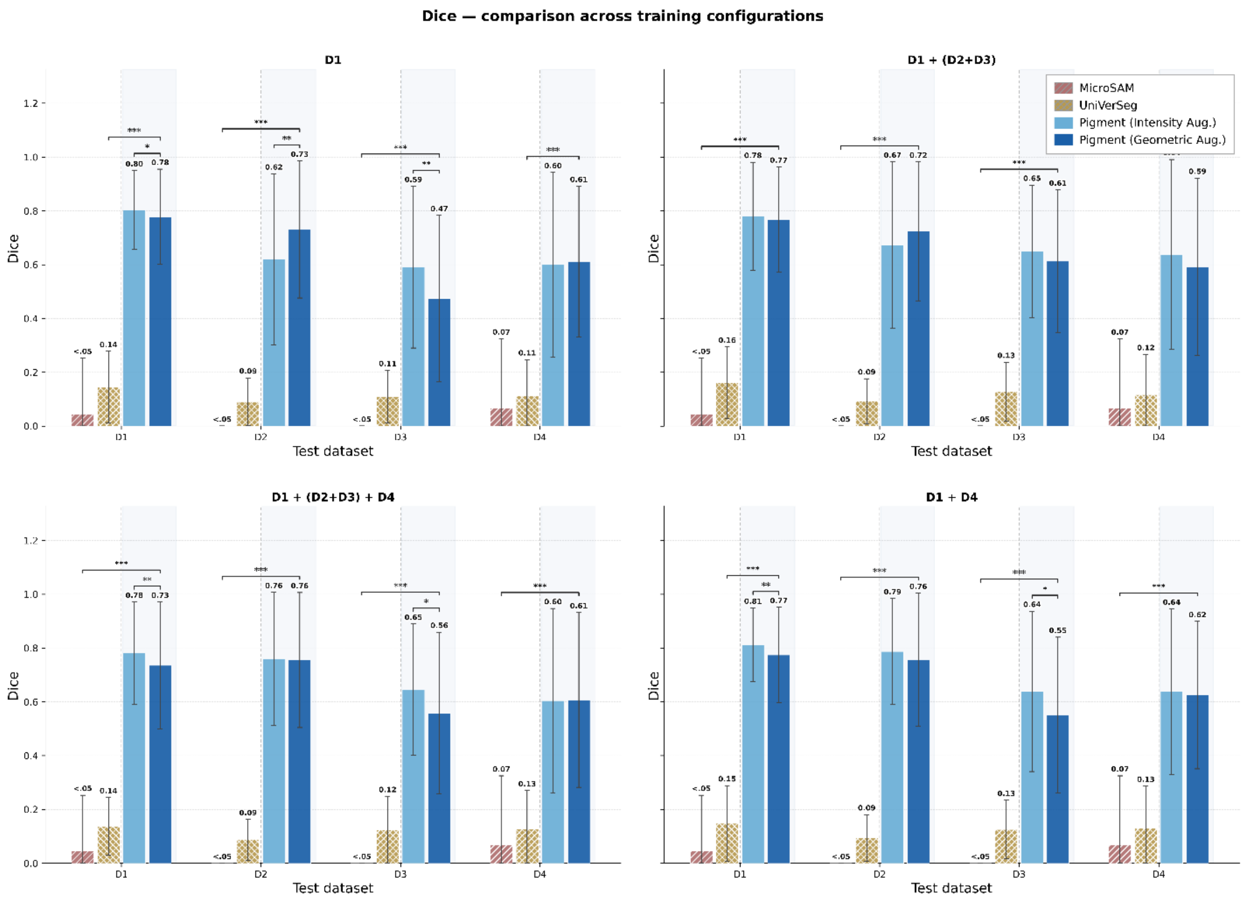

Figure S 3 Dice across test sections and training configurations for MicroSAM, UniverSeg, and PIGMENT under intensity-only and full geometric augmentation. PIGMENT achieves the highest Dice in most settings.

bioRxiv preprint doi: https://doi.org/10.64898/2026.06.18.733245; this version posted June 29, 2026. The copyright holder for this preprint (which was not certified by peer review) is the author/funder, who has granted bioRxiv a license to display the preprint in perpetuity. It is made available under a CC-BY 4.0 International license.

Fig. S4.

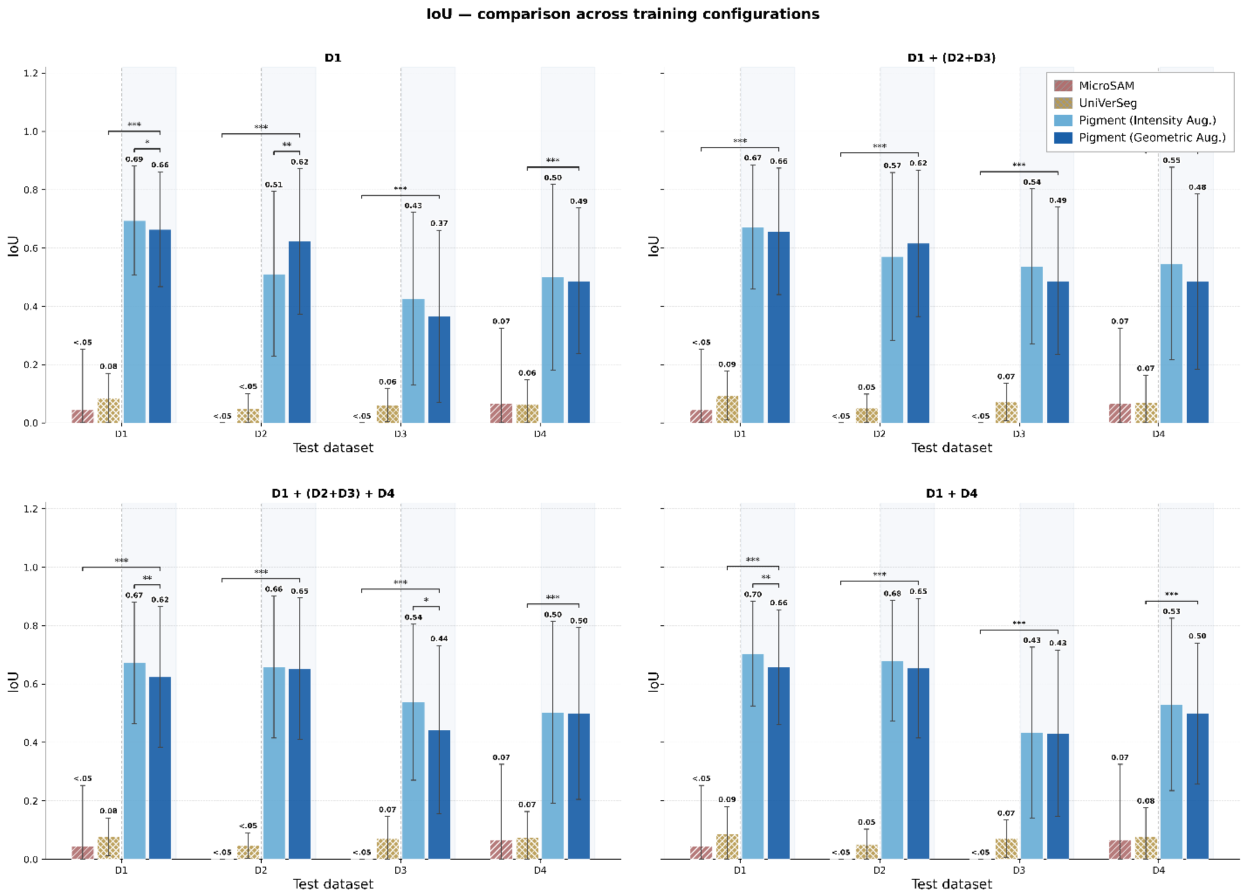

Figure S 4 Intersection-over-union (IoU) across test sections and training configurations for MicroSAM, UniverSeg, and PIGMENT. PIGMENT achieves the highest IoU in most settings, consistent with the Dice and DR comparisons.

bioRxiv preprint doi: https://doi.org/10.64898/2026.06.18.733245; this version posted June 29, 2026. The copyright holder for this preprint (which was not certified by peer review) is the author/funder, who has granted bioRxiv a license to display the preprint in perpetuity. It is made available under a CC-BY 4.0 International license.

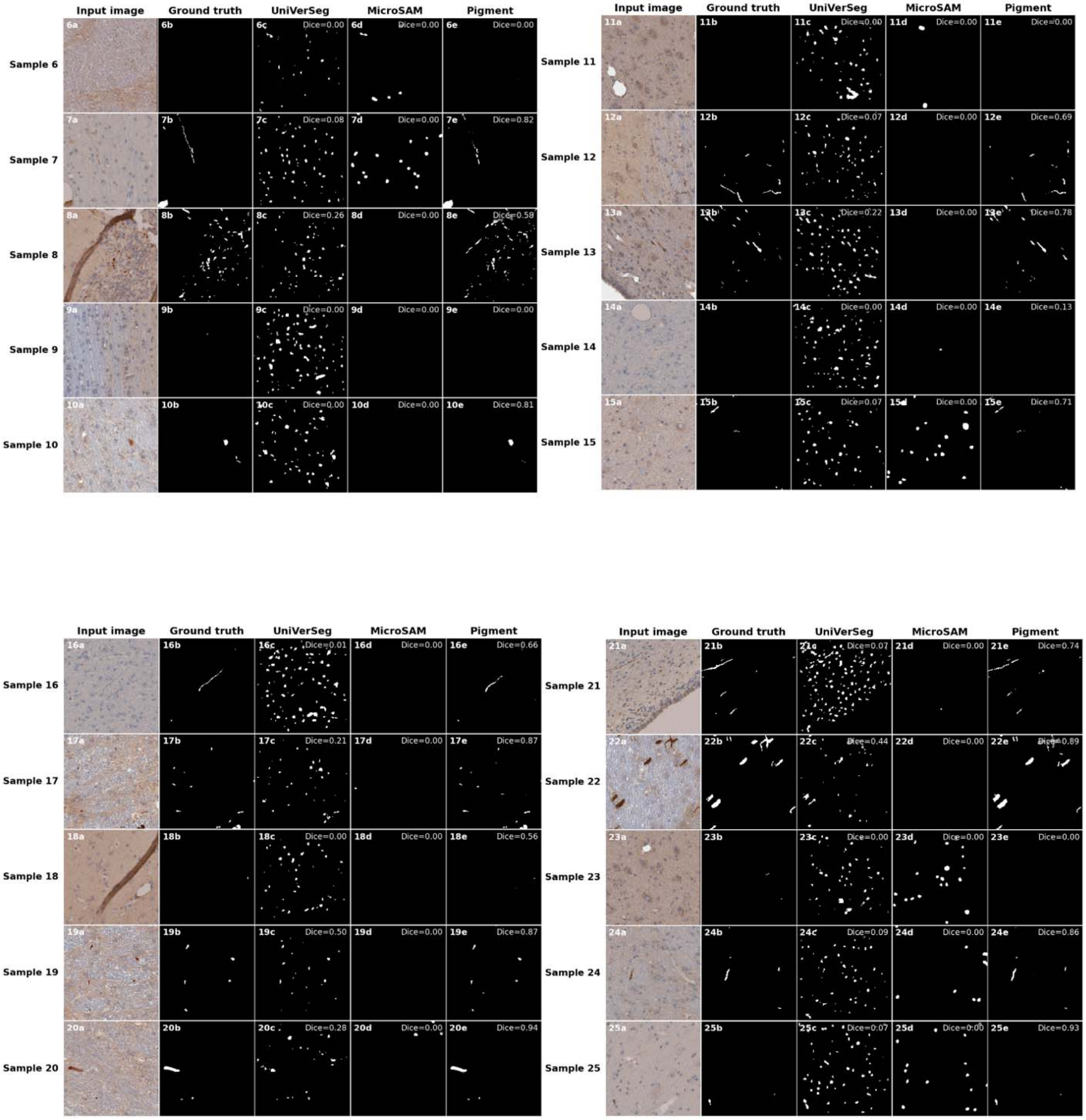

Fig. S5.

Figure S 5 Extended qualitative comparison across diverse APP morphologies, staining conditions, and annotation regimes (Samples 6-15). Representative held-out histology tiles complementing Fig. 5. Each row shows input tile, expert ground-truth annotation, UniverSeg prediction, MicroSAM prediction, and PIGMENT prediction. These supplementary samples span APP-negative windows, sparse isolated deposits, elongated axonal fibers, fragmented annotations, dense heterogeneous APP regions, and low-contrast cases. UniverSeg over-segments background tissue with fragmented false positives, and MicroSAM under-segments or fails entirely in sparse and elongated morphologies.

bioRxiv preprint doi: https://doi.org/10.64898/2026.06.18.733245; this version posted June 29, 2026. The copyright holder for this preprint (which was not certified by peer review) is the author/funder, who has granted bioRxiv a license to display the preprint in perpetuity. It is made available under a CC-BY 4.0 International license.

Mathematical formulations.

This section provides the complete mathematical specification of the components summarized in the main text Materials and Methods: the SegFormer-B0 encoder–decoder, the training objective and EMA teacher regularization, the target-aware augmentation transforms, and the post-processing applied before evaluation. Equations are numbered S1, S2, … for ease of reference.

Architecture details.

The segmentation model is a function fθ: $\mathbb{R}^{□\times W\times 3} \to [0, 1]^{□\times W}$ that maps an RGB tile of size H × W to a pixel-wise probability of APP positivity. We instantiate fθ with SegFormer-B0, a hierarchical vision transformer for semantic segmentation. The encoder (MiT-B0) is composed of four stages; at each stage, features F□ are produced by an encoder block E□ applied to the previous feature map, with spatial resolution progressively halved and channel dimensionality increased (C1 = 32, C2 = 64, C3 = 160, C4 = 256):

$$F = E(F_{-1}), \quad F_0 = I, \quad F_{+1} = PatchMerge(F). \qquad \text{(S1)}$$

Query, key, and value matrices are obtained via linear projections of the input feature matrix X with learnable projection matrices W_Q, W_K, W_V:

$$Q = X\,W\_Q, \quad K = X\,W\_K, \quad V = X\,W\_V. \qquad \text{(S2)}$$

Self-attention at each stage uses Efficient Self-Attention with a sequence-reduction ratio R□, reducing the effective key–value length from N = H□W□ to N / R□ and lowering attention complexity from $O(N^2)$ to $O(N^2 / R□)$:

$$Attention(Q, K, V) = Softmax(QK \,/\, \sqrt{}d\_\{head\})\,V, \qquad \text{(S3)}$$

where d_{head} is the head dimension. Positional information is injected via Mix-FFN blocks that combine a depthwise 3 × 3 convolution with two linear projections and a GELU non-linearity:

$$x\_\{out\} = MLP(GELU(Conv3\times 3(MLP(x)))). \qquad \text{(S4)}$$

The All-MLP decoder upsamples each encoder feature map F□ to a common spatial resolution, channel-concatenates the four upsampled maps, projects them with a linear layer, and bilinearly upsamples the logits to the input resolution:

$$M = Linear(Concat(Upsample(F_1), \ldots, Upsample(F_4))), \quad p(x) = Softmax(M(x)). \qquad (S5)$$

The model contains approximately 3.7 million parameters. The multi-section training strategy is architecture-agnostic and can be combined with any encoder–decoder segmentation network.

Training objective and EMA teacher.

Let V = {x : L(x) ≠ 255} denote the set of valid pixels, that is, pixels not marked as ignore. The training objective is pixel-wise cross-entropy computed only over V:

bioRxiv preprint doi: https://doi.org/10.64898/2026.06.18.733245; this version posted June 29, 2026. The copyright holder for this preprint (which was not certified by peer review) is the author/funder, who has granted bioRxiv a license to display the preprint in perpetuity. It is made available under a CC-BY 4.0 International license.

$$\mathcal{L}(\theta) = -(1 / |V|) \sum x \in V \sum c \in \{0,1\}\, yc(x) \log pc(x), \qquad \text{(S6)}$$

where yc(x) ∈ {0, 1} is the one-hot ground-truth label for class c and pc(x) is the predicted probability from a channel-wise Softmax on the upsampled logits. No explicit class weighting is applied; class imbalance is addressed through the augmentation pipeline.

A teacher network with parameters θ[illegible] is maintained as an exponential moving average of the student parameters θ[illegible] with decay d = 0.999. The teacher receives no gradient updates and is refreshed at every step according to

$$\bar{\theta} = d \cdot \bar{\theta}_{-1} + (1 - d) \cdot \theta. \qquad \text{(S7)}$$

The teacher flags pixel x as ambiguous (and excludes it from the gradient for that step) when its prediction strongly disagrees with the manual label in a high-confidence region:

$$ambiguous(x) = 1[L(x) \neq 255] \cdot (1[L(x) = 0 \wedge \bar{p}_1(x) > \tau_+] + 1[L(x) = 1 \wedge \bar{p}_1(x) < 1 - \tau_-]), \qquad \text{(S8)}$$

with $\tau_+ = \tau_- = 0.97$ and p[illegible]$_1$(x) the teacher's class-1 (APP-positive) probability at pixel x. Pixels flagged ambiguous in a given step are temporarily assigned the ignore value 255 and excluded from the cross-entropy loss for that step. This is a Mean-Teacher-style regularization that reduces the influence of likely mis-annotated pixels (faint deposits missed by the annotator, boundary pixels of small fragmented components, or staining artifacts inadvertently included in foreground).

Models are optimized with AdamW (learning rate $\eta = 1 \times 10^{-}$[illegible], $\beta_1 = 0.9$, $\beta_2 = 0.999$, weight decay $\lambda = 0.01$), batch size 8, with dropout applied to encoder hidden states (p = 0.1), attention weights (p = 0.1), and the classifier head (p = 0.3).

Augmentation transforms (formal definitions).

Data augmentation is formulated as a stochastic transformation T sampled from a distribution P(τ) that acts jointly on the image–mask pair (I, L):

$$(I', L') = T(I, L), \quad T \sim P(\tau). \qquad \text{(S9)}$$

The model is trained to minimize the expected cross-entropy loss over augmented samples, where D is the dataset distribution:

$$min\theta\ E(I,L) \sim D\ ET \sim P(\tau)\ [\mathcal{L}(f\theta(T(I)), T(L))]. \qquad \text{(S10)}$$

For copy-paste operations, APP-positive components are sampled only from the ground-truth mask of the same training image, so that no labels are transferred across images. Spatial transformations act jointly on image and mask to preserve pixel-wise correspondence; photometric transformations act on the image only. Pixels assigned the ignore label 255 are preserved through all transforms and excluded from loss computation. Below, x denotes a 2D spatial coordinate inside the tile and D the tile width.

Affine transform. Each spatial location x is mapped according to

$$\varphi\_\{aff\}(x) = s \cdot R(\theta) \cdot x + t, \qquad \text{(S11)}$$

bioRxiv preprint doi: https://doi.org/10.64898/2026.06.18.733245; this version posted June 29, 2026. The copyright holder for this preprint (which was not certified by peer review) is the author/funder, who has granted bioRxiv a license to display the preprint in perpetuity. It is made available under a CC-BY 4.0 International license.

where R(θ) is a 2×2 rotation matrix with θ ~ U(−18°, 18°), scale s ~ U(0.96, 1.06), and translation t_{x}, t_{y} ~ U(−0.06 D, 0.06 D). Image pixels are resampled with bilinear interpolation; masks with nearest-neighbour interpolation.

Global RGB shift. Each colour channel c ∈ {R, G, B} is shifted by an additive offset

$$I'(x;\,c) = I(x;\,c) + \Delta(c), with\ \Delta(R), \Delta(B) \sim U(-8, 8)\ and\ \Delta(G) \sim U(-6, 6), \qquad \text{(S12)}$$

in the [0, 255] pixel range. The narrower green-channel range reflects its greater sensitivity to APP immunoreactivity in brightfield histology.

Multi-instance copy-paste. Let $C_{\square}$ denote the k-th sampled connected component from L. Each instance is rigidly transformed,

$$C_{\square}' = s_{\square} \cdot R(\theta_{\square}) \cdot C_{\square} + d_{\square}, \qquad \text{(S13)}$$

with n ~ U({5, …, 20}) instances per image, scale $s_{\square}$ ~ U(0.90, 1.25), in-plane rotation $\theta_{\square}$ ~ U(−8°, 8°), and placement offset $d_{\square}$ sampled uniformly over the tile. An optional elastic deformation with amplitude α ~ U(0, 180) and smoothness σ ~ U(0, 5) is applied to introduce sub-pixel shape variation, and a 2-pixel feather blend is applied at the component boundary to suppress visible seams.

GMM-guided label promotion. A Gaussian Mixture Model with K = 4 components is fitted to the RGB distribution of manually annotated APP-positive pixels. For a pixel x with colour vector I(x), the likelihood under the mixture is

$$p(I(x)) = \sum_{k=1} K\ \pi k \cdot N(I(x);\ \mu k,\ \Sigma k), \qquad \text{(S14)}$$

and the posterior probability that pixel x belongs to mixture component k is

$$\gamma_{\square}(x) = \pi_{\square}\ N(I(x);\ \mu_{\square},\ \Sigma_{\square}) \;/\; \sum_{\square=1} K\ \pi_{\square}\ N(I(x);\ \mu_{\square},\ \Sigma_{\square}). \qquad \text{(S15)}$$

Pixels whose likelihood exceeds a data-driven threshold and that lie within a bounded ROI (at most 224 × 224 pixels, at most 3 candidate regions, with no more than 4500 promoted pixels per image) are promoted to foreground. The strict spatial and count constraints prevent promotion of false positives in regions of clearly negative tissue.

Ground-truth neighborhood diffusion. The binary mask L is convolved with a Gaussian kernel of radius r ~ U(4, 10) pixels to produce a soft proximity map L̃. A blended image Ĩ is formed as

$$\tilde{I}(x) = I(x) + \alpha \cdot \tilde{L}(x) \cdot (I\_[29] - I(x)), \quad \alpha \sim U(0.12, 0.28), \qquad \text{(S16)}$$

where I_[29] is the mean intensity of APP-positive pixels. The blend is gated by the condition L̃(x) ≥ τ, with τ ~ U(0.75, 0.90), restricting the modification to regions immediately adjacent to annotated foreground and preventing label spread into clearly negative tissue. A complete listing of transform categories and hyperparameters is given in Table S2.

Post-processing and evaluation metrics (formal definitions).

Morphological post-processing of predictions. For each connected component $C_{\square}$ in the predicted binary mask, the minimum bounding rectangle yields principal-axis dimensions ($w_{\square}$, $h_{\square}$). The elongation ratio is

bioRxiv preprint doi: https://doi.org/10.64898/2026.06.18.733245; this version posted June 29, 2026. The copyright holder for this preprint (which was not certified by peer review) is the author/funder, who has granted bioRxiv a license to display the preprint in perpetuity. It is made available under a CC-BY 4.0 International license.

$$\varepsilon_{\square} = \max(w_{\square}, h_{\square}) / (\min(w_{\square}, h_{\square}) + \varepsilon), \qquad \text{(S17)}$$

with $\varepsilon = 10^{-\square}$ for numerical stability. Components satisfying both $\text{area}(C_{\square}) \geq \tau_{area}$ and $\varepsilon_{\square} \geq \tau_{\varepsilon}$ are suppressed (set to background); we use $\tau_{area} = 2300$ pixels and $\tau_{\varepsilon} = 3.0$. Thresholds were chosen so that no annotated APP-positive component in the training set is excluded, and structures of this scale and shape correspond to staining artifacts at fold edges or tissue tears rather than APP-positive profiles.

Boundary-aware ignore masking. For each connected component $C_{\square}^{GT}$ in the binary ground-truth mask, the component is excluded from evaluation if it both (i) intersects the patch border, i.e. there exists $x \in C_{\square}^{GT}$ with $x \in \partial\Omega$, and (ii) has area below a threshold, $\text{area}(C_{\square}^{GT}) < \tau_{edge}$ with $\tau_{edge} = 50$ pixels. The resulting ignore mask $M_{[30]}$ is applied to both ground truth and prediction before metric computation:

$$g'(x) = g(x) \cdot 1[x \notin M_{[30]}], \quad p'(x) = p(x) \cdot 1[x \notin M_{[30]}]. \qquad \text{(S18)}$$

Detection rate construction. Small gaps within the GT foreground are first closed using morphological closing with a disk-shaped structuring element $B_{\square}$ of radius $r = 2$:

$$G' = (G \oplus B_{\square}) \ominus B_{\square}. \qquad \text{(S19)}$$

The predicted mask is dilated by $B_{\square}'$ with $r' = 1$ to introduce a small spatial tolerance, $P' = P \oplus B_{\square}'$, and predicted components with area below $\tau_{\square\square} = 25$ pixels are discarded. A ground-truth component $C_{\square}^{GT}$ is considered valid if $\text{area}(C_{\square}^{GT}) \geq \tau_{\square\square} = 25$ pixels and it has not been excluded by the boundary-aware ignore mask. A valid component is detected if

$$\text{detected}(C_{\square}^{GT}) = 1[\exists\, x \in C_{\square}^{GT} : P'(x) = 1]. \qquad \text{(S20)}$$

The global detection rate (Eq. 2 in the main text) is computed by summing detections over all test tiles and dividing by the total number of valid components; here we have reproduced the construction in full to make the relationship between the morphological tolerances (r, r') and the metric explicit.

Ground-truth binarization.

The annotator's overlay was rendered as a distinct green channel; after luminance conversion to grayscale, the annotation pixels were separated from background tissue by an intensity threshold (pixels with grayscale intensity at least 228 were assigned APP-positive, pixels at most 227 were assigned background). The threshold was determined empirically on representative training tiles and verified to reproduce the annotator's intent. PIGMENT consumes the resulting binary masks directly.
